# Supplementary material for: Delirium due to hip fracture is associated with activated immune-inflammatory pathways and a reduction in negative immunoregulatory mechanisms
Source: BMC Psychiatry. 2022 May 31;22:369. doi: 10.1186/s12888-022-04021-y (PMC9158285; doi:10.1186/s12888-022-04021-y)
Supplement: Supplementary file 1 — Additional file 1: Figure S1. Study flowchart. [file 12888_2022_4021_MOESM1_ESM.docx]

**Supplementary file 1**

Delirium due to hip fracture is associated with activated immune-inflammatory pathways and a reduction in negative immunoregulatory mechanisms

Paul Thisayakorn MD ^1^, Yanin Thipakorn MD ^2^, Saran Tantavisut MD PhD ^3^, Sunee Sirivichayakul Ph.D. ^4^, Michael Maes, MD Ph.D ^2,5,6^

1. Department of Psychiatry, Hip Fracture Research Unit, Faculty of Medicine, Chulalongkorn University, Bangkok, Thailand
2. Department of Psychiatry, Faculty of Medicine, Chulalongkorn University, Bangkok, Thailand
3. Department of Orthopedics, Hip Fracture Research Unit, Faculty of Medicine, Chulalongkorn University, Bangkok, Thailand
4. Department of Medicine, Faculty of Medicine, Chulalongkorn University, Bangkok Thailand
5. Department of Psychiatry, Medical University of Plovdiv, Plovdiv, Bulgaria.
6. IMPACT Strategic Research Center, Deakin University, Geelong, Australia.

Corresponding authors:

Dr. Paul Thisayakorn, M.D.

Prof. Dr. Michael Maes, M.D., Ph.D.

Department of Psychiatry

Faculty of Medicine

Chulalongkorn University

Bangkok

Thailand

[(14) Michael Maes | Stats (researchgate.net)](https://www.researchgate.net/profile/Michael-Maes-5/stats)


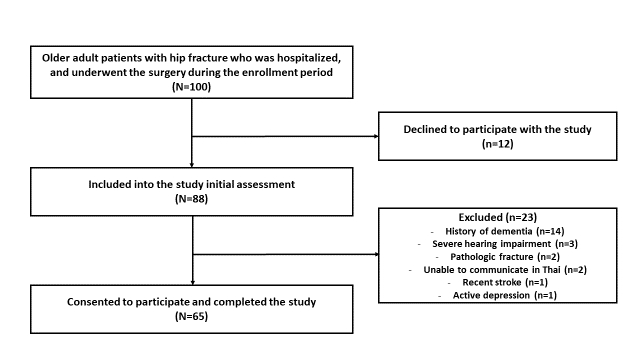


**Figure S1** Study flowchart
